# Research progress of vagus nerve stimulation in the treatment of epilepsy

**DOI:** 10.1111/cns.13209

**Published:** 2019-08-19

**Authors:** Jing‐Jing Fan, Wei Shan, Jian‐Ping Wu, Qun Wang

**Affiliations:** ^1^ Department of Neurology, Beijing Tiantan Hospital Capital Medical University Beijing China; ^2^ National Clinical Research Center for Medicine of Neurological Diseases Beijing China; ^3^ Beijing Institute for Brain Disorders Beijing China; ^4^ Advanced Innovation Center for Human Brain Protection Capital Medical University Beijing China

**Keywords:** drug‐resistant epilepsy, epilepsy, neuromodulation, vagus nerve stimulation

## Abstract

The International League Against Epilepsy (ILAE) defined drug‐resistant epilepsy (DRE) that epilepsy seizure symptoms cannot be controlled with two well‐tolerated and appropriately chosen antiepileptic drugs, whether they are given as monotherapy or in combination. According to the WHO reports, there is about 30%‐40% of epilepsy patients belong to DRE. These patients need some treatments other than drugs, such as epilepsy surgery, and neuromodulation treatment. Traditional surgical approaches may be limited by the patient's clinical status, pathological tissue location, or overall prognosis. Thus, neuromodulation is an alternative choice to control their symptoms. Vagus nerve stimulation (VNS) is one of the neuromodulation methods clinically, which have been approved by the Food and Drug Administration (FDA). In this review, we systematically describe the clinical application, clinical effects, possible antiepileptic mechanisms, and future research directions of VNS for epilepsy.

## 1 INTRODUCTION

Epilepsy is a chronic disease characterized by sudden abnormal discharge of brain neurons, which leads to transient brain dysfunction and is defined as the presence of spontaneous recurrent seizures. The lifetime prevalence of epilepsy is estimated at 1%‐5% globally.^1^ It becomes the second most common disease after headache in neurology department all over the world.^2^

The treatment of epilepsy includes drugs, surgery, neuromodulation, and ketogenic diet. According to Martin's research,^3^ more than 30% of epilepsy patients are drug‐resistant epilepsy (DRE). The International League Against Epilepsy (ILAE) defined that DRE is uncontrollable seizure within two well‐tolerated and appropriately chosen antiepileptic drugs, whether they are given as monotherapy or in combination.^4^ Especially, focal epilepsy such as temporal lobe epilepsy and some epilepsy syndromes such as Lennox‐Gastaut syndrome, West syndrome, and O'Hara syndrome could not be wholly controlled by drugs. Surgical resection is one of the choices of epilepsy treatments, but not all patients meet the criteria for the surgical procedure. Even though the epileptogenic focus is removed surgically, 30%‐50% of the patients cannot become seizure‐free.^5^ Therefore, in addition to drugs and surgery, there should be an alternative way for epilepsy patients to reduce their epilepsy syndrome and sufferings.

## 2 THE VAGUS NERVE STIMULATION

In the past few years, neurostimulation has been proved to be a safe and effective method in many preclinical and clinical trials, which can be combined with traditional drug therapy to reduce seizures. At present, the American FDA has approved vagus nerve stimulation (VNS) for the treatment of epilepsy.^6^, ^7^, ^8^ In this study, we mainly focus on some evidence‐based medical evidence related to VNS.

Vagus nerve stimulation is a relatively novel therapy method in the management of the neurological disease such as depression, epilepsy, tinnitus, and schizophrenia.^9^, ^10^, ^11^ At present, there are many studies on the treatment of epilepsy and depression, and the effect is more affirmative. There is insufficient evidence for the treatment of schizophrenia and tinnitus by VNS. It was first proposed in the 1880s in Ref.^12^ Nothnagle considers that “venous hyperaemia of the brain” caused by carotid artery pulsation leads to seizures. Electrical stimulation of the vagus nerve may reduce the “venous hyperaemia of the brain.” According to the theory, James Corning developed several carotid artery compression devices for the treatment of seizure. He combined the instrumented carotid artery compression with transcutaneous vagal nerve stimulation device to decrease cerebral blood flow. He was the first physician to report the usage of transcutaneous electrical stimulation of the vagus to interrupt convulsions in 1883.^13^ After a century of exploration, in 1988, it was first reported that the VNS device was implanted into the human body for the treatment of drug‐resistant epilepsy with chronic VNS.^14^ Vagus nerve stimulation was approved as an adjunct therapy to reduce the frequency of seizure aged ≥12 years with DRE by the American FDA in 1997.^15^, ^16^ In 2017, the FDA approved of using VNS in patients over 4 years old that characterized partial seizure and intractable epilepsy.^17^ Vagus nerve stimulation also has evidence for treatment of depression, which was approved by the FDA and may be useful for other comorbidities of epilepsy.^18^

## 3 APPLICATION

Indeed, many clinical studies have proved VNS is safe and effective in the treatment of epilepsy. A multicenter controlled study of VNS in the treatment of focal epilepsy (EO3) recruited 67 patients who had completed the blinded acute phase (14‐weeks) and properly randomized analysis. One group (N = 31) was treated with high‐frequency stimulation (0.25‐3.0 mA, 20‐50 Hz, 500 μs), and the control group (N = 36) was treated with low‐frequency stimulation (0.25‐3.0 mA, 1‐2 Hz, 130 μs). After 14 weeks of VNS treatment, the result showed that the mean seizure frequency reduced about 30.9% in the high‐frequency stimulation group. However, in the low‐frequency stimulation group it was about 11.3%. There was a statistical difference (*P* = .029) in the change of seizure frequency between these two groups. In the high‐frequency stimulation group, the frequency of seizure decreased by more than 50% is about 39%, while in the low‐frequency stimulation VNS group, it is about 19%. In the aspect of 50% reduction, the comparison of these two groups did not reach the statistical significance requirement (*P* = .0704), but there is a big tendency between these two groups. Although it has been reported that the intensity and severity of seizures have been reduced in individual patients, no significant statistical difference has been found for the time being.^15^ Despite recruited a small number of subjects and performed short‐time follow‐up study, this study lays a foundation for the wide application of the VNS.

Some other randomized controlled clinical trials have also confirmed similar findings.^15^, ^19^, ^20^, ^21^ A retrospective study from our hospital also proved that 60 out of 94 patients achieved the therapeutic effect (50% frequency of seizure decreased) from November 2008 to April 2014, regardless of age and gender.^22^ To confirm long‐time treatment safety and understand the efficacy of time cumulation, a single‐center study of VNS treatment for epilepsy followed up for 10‐17 years (N = 74) showed that the rates of seizure frequency reduction between 50% and 90% were 38.4%, 51.4%, 63.6%, and 77.8% in the years of 1, 2, 10, and 17 after VNS treatment, respectively. The rates of seizure frequency reduction more than 90% were 1.4%, 5.6%, 15.1%, and 11.1% in corresponding treatment follow‐up time.^23^ With the prolongation of treatment, the VNS therapeutic effect is gradually enhanced.

Although the FDA has approved VNS for intractable epilepsy in children and adults, the application of VNS in epilepsy remains controversial due to its small sample size and short follow‐up time. To further demonstrate the efficacy of VNS and identify which patient populations respond best to treatment, Englot et al conducted a large retrospectively registry‐based study. In this VNS treatment study, totally about 1285 physicians from 978 centers registered outcomes of 4483 patients treated with VNS.^24^ Compared with the patients' ages, disease course, and seizures types and followed about 1 year, more than half of the patients responded to VNS treatment (with a 50% reduction in seizure frequency). First, the younger patients under 18 years old (60% fewer seizure frequency than baseline) respond better to VNS treatment than those over 18 years old (53% fewer seizure frequency than baseline). Studies have proved that children may get more benefit from the VNS treatment compared with adults group. Second, the course of epilepsy before VNS device implantation is a potential predictor for the response to VNS treatment. Less than 10 years' seizure history is predicted a somewhat higher clinical response (56% vs 52%) to VNS (OR, 1.19; 95% CI, 1.00‐1.41). The result suggests that individuals with shorter epilepsy course are more favorable to respond to VNS. Therefore, the earlier the VNS treatment intervention, the better the efficacy may be. Third, according to the types of seizures, the individuals with predominantly focal seizures, including auras, achieved the most significant clinical benefit. These patients were more likely to respond to VNS than other types of seizures by 1 year's study (OR, 1.37; 95% CI, 1.04‐1.81, *P* = .025).

The therapeutic effect of VNS may not immediately respond to the stimulation, and the frequency of seizure begins to decrease after implantation within a few months. During this period, some patients may change the dosage of antiepileptic drugs due to their displeasure for the seizure control, and these changes may have an impact on the evaluation of the efficacy of VNS. To clarify this controversy, Garcia‐Pallero et al recruited 85 patients undergoing VNS operation who were included for prospective analysis and followed up after the operation. Among them, 43 patients were not allowed to change antiepileptic drugs during the follow‐up period, and 42 patients could change antiepileptic drugs under the guidance of doctors. Comparing these two groups following within 18 months, the results showed that 54.1% of the patients had more than 50% frequency of seizure reduction. About 63% of the patients in drug unchanged group had a 50% frequency of seizure reduction, and 45.2% of the patients within drug change group had the 50% frequency of seizure reduction. Therefore, there is no statistical difference in frequency reduction whether there is any change of drugs.^25^ Changing the dosage and type of antiepileptic drugs (AED) may not influence the treatment results of VNS.

In the treatment of epilepsy including children and adults, VNS treatment has been proved safe and effective.^26^ Thus, the FDA has approved that VNS can be used in children over 4 years old recently.^27^, ^28^, ^29^, ^30^, ^31^ The VNS treatments do not only reduce the frequency of seizures but also reduce the psychological burden of children.^32^ There is another study has reported that VNS may have a therapeutic effect on epilepsy patients <3 years old.^33^ In this clinical research, the authors concluded that the frequency of status epilepticus was also significantly reduced after VNS treatment.^33^ However, these studies have a small sample size within a short follow‐up. There are no other large randomized controlled trials to provide evidence‐based medical research related to the child.

Vagus nerve stimulation treatment may also have therapeutic effects on epilepsy of some individual etiology or epilepsy syndrome, such as the tuberous sclerosis complex (TSC),^34^ Lennox‐Gastaut syndrome (LGS),^35^ generalized epilepsy with febrile seizures plus (GEFS+),^36^ absence epilepsy,^37^ status epilepticus (SE),^38^ reflex eating seizures,^39^ and startle‐induced seizures.^40^ Grioni and Landi^34^ followed up 4 DRE patients with tuberous sclerosis complex (TSC) treated with VNS. The results showed that 3 out of four patients reached class IA (McHugh) and the last one patient reached class IIA (McHugh). It was worth to clarify that one patient reached seizure free without medications at the last follow‐up days (a mean follow‐up of 7 years, range 4‐12 years). The severity seizures in one patient were reduced. The patient had only brief seizures with impairment of awareness (used to present with drop attacks). Two patients stopped using antiepileptic drugs after VNS completely controlled epilepsy with a mean follow‐up of 7 years. Vagus nerve stimulation itself seems to be able to control seizures. They believe that once the stimulation effect is stable, drugs should be withdrawn as soon as possible. However, there is no evidence from large‐scale clinical trials to conclude this, and more evidence is needed to address this issue in the near future.

The results show that VNS can also improve the comorbidity of epilepsy, such as mood disorders and cognitive deficits. These are two large pivotal clinical trials confirmed the effectiveness of VNS in the treatment of refractory partial epilepsy and also concluded that epilepsy patients who had comorbid depression improved.^15^, ^19^, ^20^, ^41^, ^42^ Since then, more and more studies have been conducted on the treatment of depression with VNS treatment. It is effective in patients with treatment‐resistant depression (TRD). Treatment‐resistant depression is an American FDA‐approved indication for VNS.^43^

The overall effect of VNS on cognition is unclear, and many studies focus on memory. There is an evidence that VNS treatment can acutely improve memory in a short time, but there seems to be no significant improvement within the long‐term treatment.^44^, ^45^, ^46^ Orosz et al^31^ surveyed the effects of VNS treatment on 347 pediatric patients, evaluating the different aspects of cognition such as memory, concentration, alertness, language communication, and academic progress at 12 and 24 months. The results showed that between 12 and 24 months, a relatively high proportion of patients improved significantly in attention, language communication, and academic progress, while about half of the patients did not show an improvement in memory.

## 4 THE MECHANISMS ABOUT VNS REGULATION

Although the therapeutic effect of VNS is remarkable in the clinic, the mechanism of VNS treatment in epilepsy is not completely understood. Currently, some potential mechanisms are proposed.

Neuroelectrophysiology: Some preclinical studies are of great help in exploring VNS regulation mechanism. Early studies initially revealed that VNS treatment could cause desynchronization of cortical electrical activity in cats.^47^ Zanchetti et al^48^ found that VNS blocked the interictal spike in a cat model of epilepsy. Alexander et al^49^ found that VNS increased the threshold of amygdala seizures. Moreover, they further explored the regulation of VNS on amygdala and hippocampus at electrophysiological and protein levels.^50^ It was found that VNS modified the firing rate of amygdala neurons and increasing the intensity of VNS resulted in the number of neurons, and the firing rate was significantly affected. At the same time, the proteome of postsynaptic density (PSD) in amygdala/hippocampus neuron was detected. The proteome PSD is a kind of postsynaptic membrane‐specific located functional protein complex, which is crucial to the structure, function, and plasticity of excitatory synapses in the central nervous system. Protein composition of PSD is regulated by neuronal activity.^51^ They found that more than 425 proteins were identified in PSD comparing with those implanted but nonstimulated control group animals. Among them, 22 proteins were altered, including neurexin‐1α, and cadherin 13 and α2δ1. The increased protein content because of VNS played a role in the plasticity process of excitatory synapses. Therefore, VNS may modify neuronal activity of amygdala and hippocampus and change the composition of excitatory synapses in the central nervous system.

Neurotransmitters: Initially, studies have shown that the role of VNS in epilepsy is related to the increase in extracellular norepinephrine.^52^ Some studies have suggested that VNS can promote the release of norepinephrine from locus coeruleus, thus achieving the antiepileptic effect.^53^, ^54^, ^55^ Activation of neurons in locus coeruleus (LC) is thought to regulate many functions in the central nervous system related to antiepilepsy effect. Daniel et al used different VNS parameters to describe the neuronal response in LC. They reported that transient (0.5 seconds) VNS resulted in rapid and periodic neuronal activity in LC. Apparent phase response was observed at low (0.1 mA) stimulation intensity. Increasing current intensity and pulse width can drive greater nerve activity. The increased inhibitory gamma‐aminobutyric acid (GABA) signaling or decreased excitatory glutamate signaling within the NST reduces susceptibility to chemically induced limbic motor seizures.^56^ A study shows that VNS elevates the levels of GABA in cerebrospinal fluid.^57^

Inflammation: More and more evidence shows that inflammation and immune activation system play an essential role in the occurrence of epilepsy. Several studies have been provided that VNS treatment can lower seizure threshold, and antiinflammatory therapy may improve the prognosis of epilepsy.^58^, ^59^, ^60^, ^61^ Many studies have shown that VNS treatment can alleviate inflammation state in many inflammatory‐related diseases, such as sepsis, excessive ischemia‐reperfusion injury, and arthritis.^62^, ^63^ Therefore, it is speculated that one of the underlying mechanisms of the therapeutic effect with VNS treatment in epileptic patients may be the alleviation of inflammation. Varvel et al^64^ observed that infiltrating monocytes aggravated neuronal damage and increased the incidence after status epilepticus. This indirectly proves that improving inflammation may reduce epileptic seizures. Vagus nerve stimulation can reduce the cytokines released by innate immune cells in spleen, and may transfer macrophage phenotypes from preinflammation to reparative.^65^ It is unknown how VNS directly or indirectly regulates inflammatory factors in the central nervous system.

The VNS delivers electrical stimulation to the left cervical vagus nerve trunk, activating axons of afferent neurons, and subsequent changes to neuronal excitability throughout the central nervous system.^66^ However, the exact regulation mechanism that VNS modulates seizure activity remains unclear. The mechanism of VNS treatment could be studied in two aspects. The one is structure connection study, and the other one is the functional multiple brain connection. Structurally, it should be clear which brain regions reacted during VNS treatment period, and functionally, which neurons or transmitters or cytokines can be induced activity in these regions. And also, we should clarify its function property, inhibitory or excitatory. Many studies have focused on the neural networks associated with the vagus nerve, but there are no definite conclusions. Vagal afferents primarily project to the nucleus of the solitary tract (NTS), and the NTS, in turn, sends monosynaptic projections to different regions of the brain in modulating the activity of subcortical and cortical circuitry. Monoamine nuclei in the brainstem, the LC, and the raphe nuclei receive direct or indirect projections from the NTS.^54^, ^67^ The forebrain and limbic system also receive NTS projections, including the bed nucleus of the stria terminalis, paraventricular, dorsomedial, and arcuate hypothalamic nuclei, preoptic and periventricular thalamic nuclei, and central amygdala nucleus.^68^, ^69^ However, how these brain regions are connected and functioning is not very clear.

In the past, most of the electrophysiological methods were used to record the activities of local brain areas. Recently, many studies have applied functional neuroimaging to explore the effects of VNS which to be characterized throughout the brain. Some researchers used positron emission tomography (PET) or single‐photon emission computerized tomography (SPECT) to detect increased metabolism or blood flow in thalamus, hippocampus, amygdala, lower cerebellum, and cingulate cortex during or after VNS treatment.^70^, ^71^, ^72^, ^73^ However, these techniques are difficult to capture the dynamics of poststimulus responses because of their poor temporal resolution. Functional magnetic resonance imaging (fMRI) has a high temporal and spatial resolution, which can compensate for this shortcoming. Some VNS‐fMRI studies have reported that VNS evoked blood oxygenation level–dependent (BOLD) responses in the hypothalamus, amygdala, hippocampal formation, thalamus, and prefrontal cortex.^74^, ^75^, ^76^, ^77^, ^78^ However, the results are not always consistent.

Our team traced the neural circuits of VNS with viruses and found that they were activated in the cortex, hippocampus, and lower part of nucleus accumbens of rats. Next, we will carry out functional exploration and verification.

## 5 SAFETY AND COMPLICATIONS

Many studies have shown that VNS implantation is a relatively safe operation. It has been reported that there is no definite teratogenicity in pregnant women treated with VNS because the sample size is too small; further research is needed.^79^ The common complications include infection, postoperative hematoma, and vocal cord paralysis. The incidence rate is about 2%. Very few patients suffer from bradycardia or cardiac arrest, and most of them occur during operation. Some equipment‐related complications may also occur, such as repetitive surgery, battery replacement, lead fracture, or malfunction.^80^ Since neuromodulation has become an important treatment option of drug‐resistant epilepsy, in this review we also briefly comment on the indication, effectiveness, and adverse effects of responsive nerve stimulation (RNS) and deep brain stimulation (DBS), as compared to VNS. Detail information could be found in the following Table 1.

**Table 1:** Indication, effectiveness, and adverse effects in VNS, RNS, and ANT‐DBS

	VNS	RNS	ANT‐DBS
Indication	VNS is indicated for symptomatic localization–related epilepsy with multiple and bilateral independent foci, symptomatic generalized epilepsy with diffuse epileptogenic abnormalities, refractory idiopathic generalized epilepsy, failed intracranial epilepsy surgery, and other several reasons of contraindications to epilepsy surgery	Adults with partial‐onset seizures who have undergone diagnostic testing that localized no more than 2 epileptogenic foci and refractory to two or more antiepileptic medications, and currently have frequent and disabling seizures (motor partial seizures, complex partial seizures, and/or secondarily generalized seizures)	Bilateral stimulation of the anterior nucleus of the thalamus (ANT) for epilepsy is indicated as an adjunctive therapy for reducing the frequency of seizures in individuals 18 y of age or older diagnosed with epilepsy characterized by partial‐onset seizures with or without secondary generalization that are refractory to three or more antiepileptic medications
Effectiveness	Seizure frequency was reduced by an average of 45%, with a 36% reduction in seizures at 3‐12 mo after surgery and a 51% reduction after >1 y of therapy. At the last follow‐up, seizures were reduced by 50% or more in approximately 50% of the patients, and VNS predicted a ≥50% reduction in seizures with a main‐effects OR of 1.83	The average decrease in seizures was 44% after 1 y, 53% at 2 y, and up to 66% after 3‐6 y of using RNS. The same trend was seen when some of these people were followed for 7 y. Seizures decreased by an average of 72%	Seizures decreased on average after 3 mo by 40% in people treated with DBS as compared to only 15% for those in a placebo group (not receiving DBS). People receiving DBS for long‐term follow‐up (up to 7 y) had seizures decreased by 75%
Adverse effects	Common side effects are cough, hoarseness, voice alteration, and paresthesia. These side effects tend to diminish with time. Cognitive side effects often seen with antiepileptic drug use are not reported	Serious adverse events occurring in ≥2.5% of patients include EEG monitoring, infection, change in seizures, medical device removal, death, device lead damage or revision, antiepileptic drug toxicity, hemorrhage, psychiatric events, status epilepticus, and seizure‐related injury. Refer to the product labeling for a detailed disclosure of other reported adverse events	Serious adverse events (SAEs) accounted for 5.9% of events and device‐related SAEs were 1.7% of all events. A serious device‐related adverse event was reported in 34.5% (38/110) of subjects. There were no unanticipated adverse device effects, including death, intracranial hemorrhage, device‐related infection, and neuropsychological problem

Direct VNS is a kind of invasive surgery, which is relatively expensive, and the patients bear the risk of repetitive surgery such as battery replacement. Therefore, compared with VNS, transcutaneous vagus nerve stimulation (ta‐VNS) has many advantages, including noninvasive, price moderate, fewer side effects. Many clinical trials have proved that ta‐VNS has a similar therapeutic effect with VNS.^81^, ^82^, ^83^ Frangos et al done research that 13 healthy subjects accept cutaneous electrical stimulation to the right anterolateral surface of the neck during fMRI scanning.^84^ Compared with the control group (cutaneous electrical stimulation to the right poster‐lateral surface of the neck), transcutaneous electrical stimulation showed significant activation as the primary vagal projections, including nucleus of the solitary tract (primary central relay of vagal afferents), parabrachial area, primary sensory cortex, insula, basal ganglia, and frontal cortex. It also proved that noninvasive VNS might be as effective as invasive VNS.

## 6 CONCLUSION

Vagus nerve stimulation is an adjuvant treatment for drug‐resistant epilepsy, which had been approved to treat focal epilepsy patients more than 4 years old by the US FDA. Randomized clinical trials have provided that VNS is also a safe and effective treatment for the younger infants and children. Some clinical cases reported that VNS is also helpful for some particular epilepsy types, including tuberous sclerosis complex (TSC), Lennox‐Gastaut syndrome (LGS), generalized epilepsy with febrile seizures plus (GEFS+), absence epilepsy, status epilepticus (SE), reflex eating seizures, and startle‐induced seizures. With the prolongation of treatment, VNS can produce a cumulative effect. The antiepileptic mechanism of VNS is complex and diverse, and no one mechanism can completely explain it. It is the result of the interaction of several mechanisms. In the future, more basic science research is required to explore the structural and functional circuit of Vagus to understand the foundation mechanism and for the better application of VNS clinically.

## CONFLICT OF INTEREST

The authors declare no conflict of interest.
